# Factors affecting gasless reduced-port laparoscopic myomectomy (GRP-LM) using a subcutaneous abdominal wall lifting method: a retrospective analysis of a large cohort of 966 cases in Japan

**DOI:** 10.1007/s00404-024-07706-9

**Published:** 2024-09-09

**Authors:** Hiroe Ito, Yasukazu Sagawa, Junko Nakagawa, Tomoyoshi Akaeda, Kiyoaki Tsutsumi, Keiichi Isaka

**Affiliations:** 1 Department of Obstetrics and Gynecology, Tokyo Medical University Hospital, 6-7-1, Nishishinjuku, Shinjukuku, Tokyo, 160-0023 Japan; 2 Department of Obstetrics and Gynecology, Meirikai Tokyo Yamato Hospital, Tokyo, Japan; 3 Department of Obstetrics and Gynecology, Akaeda Clinic, Tama City, Tokyo, Japan; 4 Department of Obstetrics and Gynecology, Nagai Mothers Clinic, Misato City, Saitama, Japan; 5 Department of Gynecology, Tokyo International Ohori Hospital, Mitaka City, Tokyo, Japan

**Keywords:** Gasless surgery, Laparoscopic myomectomy, Subcutaneous abdominal wall lifting method, Reduced port surgery

## Abstract

**Objective:**

To evaluate the usefulness of gasless reduced-port laparoscopic myomectomy (GRP-LM) using a subcutaneous abdominal wall lifting method.

**Methods:**

In GRP-LM, after lifting the abdominal wall by a subcutaneous abdominal wall lifting method, a 1.5-cm incision is made in the lateral abdomen, Lap Protector^®^ is placed. The operation is performed by two surgeons, one who inserts multiple forceps from the Lap Protector and performs the operation, and an assistant who operates the laparoscope and uterine manipulator. The surgical outcome of GRP-LM and the factors that affect it were investigated.

**Results:**

GRP-LM was performed in 966 patients. Complications (0.5%) and blood transfusions (0.3%) were remarkably rare, and there were no cases of conversion to open surgery. With regard to the correlation between the number of fibroids extracted and each factor, the number of fibroids extracted correlated with fibroid weight and operation time, but not with blood loss. The average number of sutures per case was 21, and the average suture and ligation time per suture was 77 s. Comparing the cost of GRP-LM with that of the conventional insufflation LM, a saving of $875 was possible with GRP-LM.

**Conclusion:**

GRP-LM is a suitable for multiple fibroids, and is cosmetic and economical, because it allows rapid and reliable suture and ligation, despite having only one port for the procedure.

**Supplementary Information:**

The online version contains supplementary material available at 10.1007/s00404-024-07706-9.

## What does this study add to the clinical work

**Table:** 

Gasless Reduced-Port Laparoscopic Myomectomy (GRP-LM) is superior in terms of simplicity, economy, and esthetics, which are important in clinical practice, and is easy for even young physicians to adopt.	

## Introduction

Laparoscopic myomectomy (LM) has become widely used, because it is minimally invasive and cosmetically superior to open surgery. However, it is more difficult than other laparoscopic surgeries, because it requires advanced techniques, such as extensive suturing and ligation of the myometrium, traction removal of the fibroids, and removal of the fibroids from the body [1].

For this reason, it is often performed only by skilled laparoscopic surgeons. On the other hand, it has been reported that gasless laparoscopic surgery (*G*-LS) using a subcutaneous abdominal wall lifting method (SAWL) can be introduced relatively easily even by novices, because the technique of laparotomy can be applied [2–5].

Since we developed our own *G*-LS in 1993 [4], we have continued to improve it to enhance its safety, operability, economy, and cosmetic aspects. In addition, *G*-LS has advantages such as quick and reliable suturing and ligation and easy powerful traction using the Tenaculum forceps [6], and we believe that it has a great advantage in LM.

Recently, we reported on as many as 5000 cases of *G*-LS with SAWL [7]. Among them, we compared gasless reduced-port laparoscopic surgery (GRP-LS) and gasless three-port laparoscopic surgery (G3P-LS) in 1529 cases of LM, 2141 cases of laparoscopic ovarian cystectomy (LC), and 456 cases of laparoscopic salpingectomy (LT). The results showed that GRP-LS and G3P-LS had comparable outcomes in all procedures, including LM.

The purpose of this paper was to evaluate the safety, operability, and economic efficiency of this technique, as well as to examine the factors that influence this technique based on relevant patient backgrounds and pathogenesis of uterine fibroids in GRP-LM cases.

## Materials and methods

The study is a retrospective cohort study and was conducted in accordance with the ethical principles set forth in the Declaration of Helsinki and the research protocol approved by Tokyo Medical University, Institutional Review Board (Approval No. T2020-0070).

**Fig. 1:**
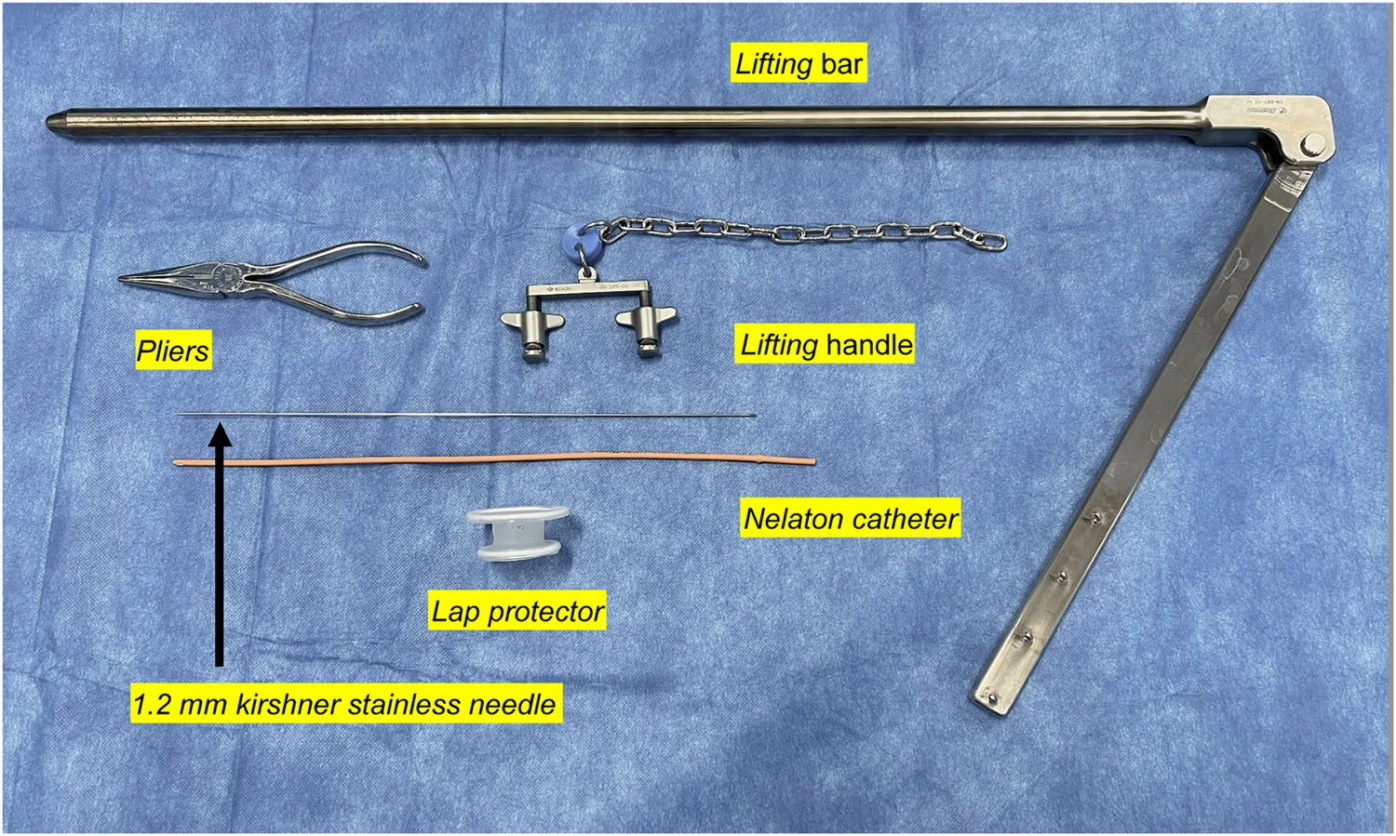
Equipment required for the subcutaneous abdominal wall lifting. They are the lifting bar, the lifting handle, the Kirschner stainless needle (1.2 mm), and the Nelaton catheter and pliers

1. Cases and surgeons
  Of the cases in which LM was performed with informed consent and consent of surgery at our hospital between January 2005 and December 2016, the currently performed GRP-LM was targeted.
  In all cases, the selection of eligible patients was based on preoperative Magnetic Resonance Imaging (MRI) and ultrasonography before the surgery was planned. Inclusion criteria for cases included a fibroid size of 10 cm or less, but any fibroid larger than this was left to the surgeon's discretion. There were no restrictions on the number of fibroids or the site of origin. On the other hand, cases with suspected uterine malignancies such as sarcomas on imaging such as MRI and severe obese cases with a body mass index (BMI) of 35 or higher were included as exclusion criteria.
  The surgeon was selected according to the difficulty of the operation, although the operation was performed by endoscopy-certified specialists, gynecologists, and residents.
2. Techniques for securing the operative field in GRP-LM
  The securing of the operative field has already been reported [7] and is briefly described here, but please refer to the supplemental files (Figure S1 and Video S1) for more details.
  SAWL was performed using the instruments from Mizuho Medical Co., Ltd (Tokyo, Japan) shown in Fig. 1. A Kirschner steel wire (1.2 mm diameter) was inserted on the subcutaneous sagittal line of the midline of the abdominal wall, and after fixing the lifting handle, the abdominal wall was fixed to the lifting arm. Then, a 1.5-cm incision was made in the right lower abdominal wall to reach the abdominal cavity, and a Lap Protector^®^ (Hakko, Chikuma, Nagano, Japan) was inserted. Next, a 5 mm trocar was inserted from the umbilical fossa under endoscopic surveillance.

Techniques for securing the operative field in GRP-LM. Supplementary file 1 (MOV 59678 kb)Suture and ligation in GRP-LM. Supplementary file 2 (MOV 85408 kb)
3. Surgical procedure in GRP-LM
  For the detailed surgical technique of GRP-LM, please refer to the supplemental file (Figure ). Here, we describe the suture ligation method specific to GRP-LM and the extracorporeal delivery of the extracted fibroid.
  3.1. Suture and ligation
    An additional movie file shows this more detail [see the Supplemental file, Video S2].
    The suture of the muscle layer was performed with a single-knot suture using CONTROL RELEASE^TM^ synthetic absorbent threads (1-0 and 3-0) or large needles (needle length 48mm, suture size 1: Vicryl JB725^® ^, Ethicon, US). The ligation was carried out by an instrumental knot, in which a knot was formed outside the body. Then, a thread was grasped and fed into the abdominal cavity for ligation. When taking out the needle, the needle was not directly grasped, but the thread was grasped several cm away from the needle. This prevented the needle from slipping near the Lap Protector.
  3.2. Removal of fibroids from the body
    For fibroids that had enucleated, threads were sewn and used as support threads to avoid straying deep inside the body. Shredding was performed by grasping a fibroid with several Kocher forceps and using a sharp-edged scalpel to cut the fibroid into pieces, as if peeling an apple.
    Currently, the excised fibroids were collected in a bag and then sectioned (Fig. 2). The Rusch MemoBag^®^ (Teleflex, Morrisville, NC) with inner diameter 100 mm was first inserted into the abdominal cavity, and then, the fibroids detained by the thread were collected in the bag. After removing the Lap Protector, the mouth of the bag was guided outside the body, the Lap Protector was reattached, and hand morcellation of the fibroids was performed using a scalpel.

**Fig. 2:**
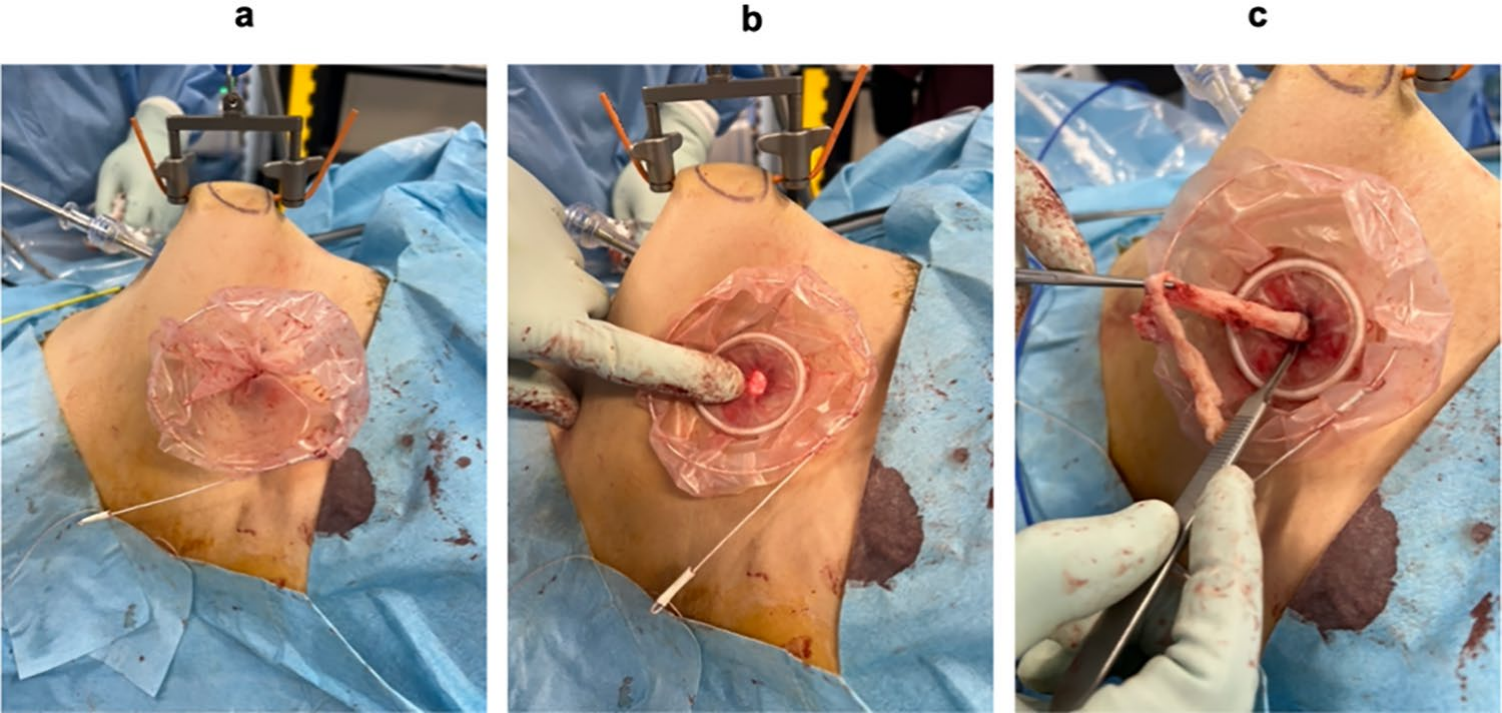
Fibroid sectioning using a MemoBag^®^. **A** Extraction of the MemoBag with fibroids retrieved out of the abdominal cavity. **B** Reattach the Lap Protector to the abdominal wall. **C** Morcellation of the fibroid using a scalpel
4. Methodology
  4.1. The patient background, operative time, blood loss, uterine fibroid weight, number of fibroids, hospital stay, complications, transition rate to laparotomy, and pathological diagnosis in GRP-LM were investigated.
  4.2. Correlations between no. of fibroids extracted, weight of fibroids, operative time, and blood loss were investigated.
  4.3. The preparation time from the start of the lifting procedures to the securing of the surgical field, as well as the number of sutures and the time per suture and ligation were examined by playing back the videos taken during the operation for 50 randomly selected cases. The size of the incision wound made in the lower abdomen was also measured postoperatively for the 20 most recent GRP-LM cases.
  4.4. To examine the economics of the procedures, we compared the prices of instruments, consumables, and other items necessary for the surgery used for GRP-LM and conventional insufflation LM at our institution.
5. Statistical analysis
  Student’s *t* test was used for comparison between two groups, and Chi-square test was used to analyze the association between the groups. The correlation between groups was performed using Pearson's product-moment correlation coefficient. The difference was judged to be statistically significant when *p* < 0.05. Statistical analysis was performed using the Statistical Package for the Social Sciences version 26.

## Results

GRP-LM was performed in 966 patients between 2005 and 2016.

1. Patient background and surgical outcomes
  The median and range of age and body mass index (BMI) of patients with GRP-LM were 37 (22–55) years and 20.6 (14.5–32.8) kg/m^2^, respectively. Eighty-nine patients (9.2% of all patients) were obese with a BMI of 25 or more. Of these, 89 patients (9.2% of all patients) had previous surgeries (Table 1).

**Table 1:** Patient background and previous surgeries in GRP-LM*

Characteristics (n** = 966)	
Age (years), median (range)	37 (22–55)
BMI*** (kg/m^2^), median (range)	20.6 (14.5–32.8)
< 18.5, n (%)	134 (13.9)
18.5 ≦ < 25.0, n (%)	743 (76.9)
25.0 ≦ < 30.0, n (%)	80 (8.3)
30.0 ≦ < 35.0, n (%)	9 (0.9)
35.0 ≦, n (%)	0 (0.0)
No. of cases with previous surgery, n (%)	89 (9.2)
Open surgery, n (%)	32 (3.3)
Cesarean section, n (%)	12 (1.2)
Laparotomy, n (%)	18 (1.9)
Multiple surgeries, n (%)	2 (0.2)
Laparoscopic surgery, n (%)	22 (2.3)
Appendectomy n (%)	35 (3.6)

*Gasless reduced-port laparoscopic myomectomy

**Number of cases

***Body mass index
  The median and range of operative time was 149.5 (30–463) minutes, blood loss was 95 (0–1930) ml, myoma weight was 150 (22–890) g, number of fibroids extracted was 4 (1–31) (mean ± standard deviation: 5.0 ± 4.3), and hospital stay was 6 (4–10) days. There were no cases of malignancy diagnosed by postoperative pathology. Complications were observed in 7 cases (0.7%), of which 1 case (0.1%) was intraoperative complication and 6 cases (0.6%) were postoperative complication. All complications were less than Grade III of the Clavien–Dindo classification. Blood transfusion was performed in three cases (0.3%), and there were no cases of laparotomy (Table 2).

**Table 2:** Surgical outcomes and complications in GRP-LM*

Surgical outcomes (n** = 966)		
Operative time (min.), median, (range)	149.5	(30–463)
Blood loss (ml), median, (range)	95	(0–1930)
Weight of fibroids extracted (g), median, (range)	150	(22–890)
No. of fbroids extracted, median (mean ± SD**), (range)	4 (5.0 ± 4.3)	(1–31)
Blood transfusion, n (%)	3 (0.3)	
Transition to open surgery, n (%)	0 (0.0)	
Hospital stay (day), median, (range)	6	(4–10)
Complication, n (%)	7 (0.7)	
Clavien-Dindo classification	Grade I and II	Grade III or higher
Intraoperative complication, n (%)	1 (0.1)	0
Intestinal injury	0	0
Subcutaneous emphysema	1	0
Lost needle	0	0
Postoperative complication, n (%)	6 (0.6)	0
Subcutaneous bleeding	4 (0.4)	0
Intrapritoneal bleeding	0	0
Infection	2 (0.2)	0
Ileus	0	0

Table 2 shows the GRP-LM surgical results, blood transfusion rate, conversion rate to laparotomy, and length of hospital stay. Intraoperative and postoperative complication rates were classified using the Clavien-Dindo classification

*Gasless reduced-port laparoscopic myomectomy

**Number of cases

***Standard deviation
2. Correlations between No. of fibroids extracted, weight of fibroids, operative time and blood loss (Table 3)

**Table 3:** Correlation between No. of fibroids extracted, weight of fibroids, operative time and blood loss

Correlation	n*	r**	*p* value
No. of fibroids versus Weight of fibroids	891	0.217	< 0.001
No. of fibroids versus Operative time	846	0.338	< 0.001
No. of fibroids versus Blood loss	910	0.147	< 0.001
Weight of fibroids versus Operative time	818	0.523	< 0.001
Weight of fibroids versus Blood loss	881	0.449	< 0.001
Operative time versus Blood loss	855	0.587	< 0.001

Pearson’s product moment correlation coefficient was used for these statistical analyses

*Number of cases

**Correlation coefficient
  An increase in the number of fibroids extracted resulted in a prolongation of operation time, an increase in blood loss, and an increase in weight of fibroids (Fig. 3). Correlations between the number of fibroids extracted and weight of fibroids, operative time, and blood loss were weakly positive for weight of fibroids and operative time, respectively, but not for blood loss (Table 3). With regard to the correlations among weight of fibroids, operative time, and blood loss, a strong positive correlation was found between each of them (Table 3). Statistics were analyzed by Pearson's product-moment correlation coefficient. To reject spurious correlation, we also examined the effect of intervening control variables using partial correlation coefficients, with similar results (data not shown in text.).

**Fig. 3:**
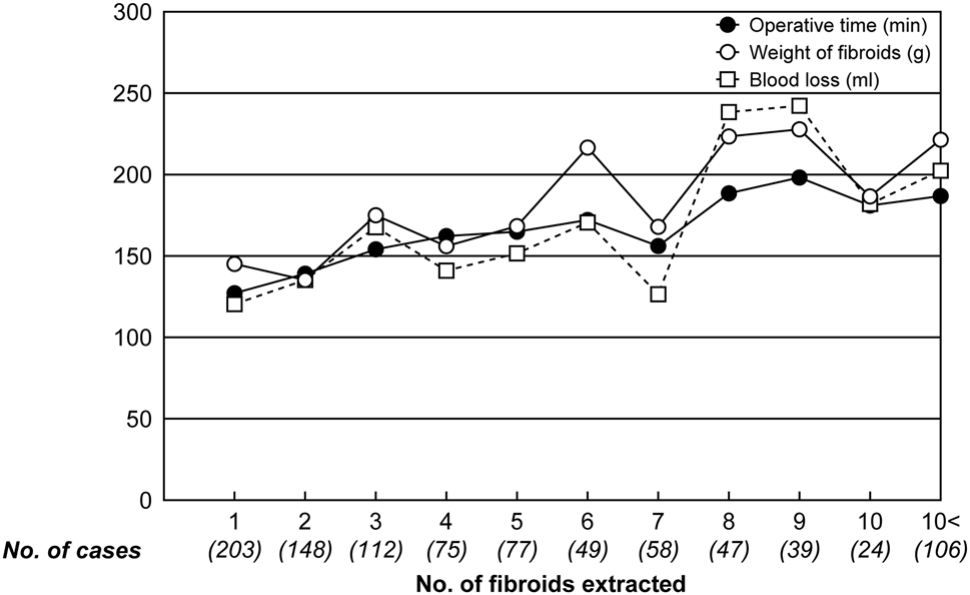
Comparison of operative time, weight of fibroids and blood loss based on No. of fibroids extracted
3. Preparation time to secure the surgical field, number of sutures per case, suture and ligation time per case, and wound size
  The preparation time (mean ± SD) from the start of the lifting operation to the securing of the surgical field and placing Lap Protector was 2.8 ± 1.4 min, and the average number of sutures per case was 21 ± 10, and the average suture time per suture was 77.4 ± 17.5 s with the posterior wall taking longer to suture than the anterior wall (Table 4). The average wound size was 1.5 ± 0.2 cm.

**Table 4:** Time required for one suture ligation (basically three ligatures)

Type of uterine layer	Site of uterine wall		*p* value
	Anterior wal	Posterior wall	
SLT* of all layers mean ± SD** (sec.), (n***)	87.5 ± 35.4 (46)	75.0 ± 28.1 (80)	0.035
SLT of myometrium mean ± SD (sec.), (n)	89.5 ± 34.2 (17)	78.9 ± 35.8 (28)	0.243
SLT of serosal layer mean ± SD (sec.), (n)	86.0 ± 36.6 (29)	73.0 ± 23.3 (52)	0.081

*One suture ligation time

**Standard deviation

***Number of suture laigation times measured
4. Sutue and ligation techniques
  The suture and ligation method in GRP-LM is clearly different from conventional LM. The advantages and disadvantages of this suture ligation are detailed in Table 5.

**Table 5:** Perceived advantages and disadvantages of suture ligation

Advantages
(1) Easy to insert and remove the needle
(2) Strong ligation is possible
(3) Large needles or controlled-release needles can be used

Disadvantages
(1) Need to reposition the needle in the abdominal cavity (to prevent tangling of threads)
(2) Special needle holder is required for suturing away from the port
(3) Frontal sutures are difficult to perform
5. Cost related to surgery
  The last 20 cases of GRP-LM and conventional insufflation LM were reviewed. Insufflation LM was a four-port technique and barbed suture was used. GRP-LM, on the other hand, used a control release suture. In-bag morcellation technique was used for extracorporeal delivery of the extracted fibroids.
  A comparison of surgery-related costs between the groups revealed a significant reduction in the cost of disposable products in the GRP-LM, mainly due to the use of only one trocar and the use of scalpels for morcellation of fibroids. As a result, the total cost was 25,274 yen for GRP-LM and 140,080 yen for conventional LM, a difference of 114,806 yen (US$875) (Table 6).

**Table 6:** Cost comparison in laparoscopic myomectomy (GRP-LM* vs. conventional LM)

Instruments and accessories	Products	Unit price	Number of use		Cost	
			GRP-LM	Conventional LM	GRP-LM	Conventional LM
Port	12 mm trocar	10,600	0	1	–	10,600
	5 mm trocar	8000	1	3	8000	24,000
	Lap Protctor^®^	5050	1	0	5050	–
Needle and thread**	Barbed suture VLoc^®^	6090	0	2	–	12,180
	Coated Vicryl^®^ control release***	3712	2	0	7424	–
Morcellation	Uterine Morcelator^®^	65,000	0	1	–	65,000
	Scalpel****	50	4	0	200	–
	Morcellator bag	24,000	0	1	–	24,000
	Menobag	4600	1	0	4600	–
Pneumoperitoneum device and gas	Tube	3100	0	1	–	3100
	CO2 gas*****	6000/cylinder	–	+	–	1200
Total cost (yen)					25,274 (a)	140,080 (b)

*Gasless reduced-port laparoscopic myomectomy

**Number used to nucleate one fibroid of about 6–7 cm

***8 strands per packet

****Average number of uses in the last 20 cases

*****Gas amount was calculated for 120 min

(b)–(a): Deduction amount = 114,806 yen ($ 875), Exchange rate (2023/03): 1 Japanese yen = 0.0076 US Dollar

## Discussion

The GRP-LM has several advantages over the conventional LM, including the ability to perform a powerful dissection by pushing with the suction tube while pulling with the Tenaculum forceps on the fibroid, and the ability to perform secure suturing and ligation (multi-layer sutures with single-knot sutures) with a needle holder familiar from open surgery. In addition, GRP-LM can be performed quickly using a controlled release needle, because suturing and ligation can be performed in a short period of time, which increases the frequency. As for the safety of using this needle, in our experience, two cases of needle loss were observed in more than 4000 cases (loss rate < 0.05%) [8]. This is comparable to the rate of needle loss during insufflation (0.07%) [9], so we believe that there is no problem if the needle is used with sufficient care to avoid loss.

As for the removal of excised fibroid, the risk of power morcellation was proposed by the United States Food and Drug Administration (US FDA) in 2015 [10]. In response to this, countermeasures are now being taken by introducing new techniques such as in-bag morcellation [11], small incision [12], wound protector–retractor [13], and vaginal approach [14]. On the other hand, it is inevitable that new problems related to complicated operations and cosmetic aspects will arise. We have been using a scalpel to make fine sections without a motorized morcellator for a long time, but now we are using a bag-based method as shown in the text. In *G*-LS, it is easy to insert the extracted fibroids into the bag, and the fine incision with a scalpel is less likely to scatter fragments, unlike morcellators. Therefore, the use of a scalpel with a bag in *G*-LS is easier than in-bag morcellation and is cosmetically superior, because it does not enlarge the wound or create new small incisions.

GRP-LM is a technique that can solve the problems of LM, such as traction of the fibroid, suture ligation of the muscle layer after removal, and removal of the fibroid.

We believe that comparisons regarding operative time, blood loss, and extracted fibroid weight showed comparable results to those of conventional LM. However, it is difficult to compare the results of this surgery, such as surgical time and blood loss, with those of conventional insufflation LM because of biases such as differences in surgical techniques and the number of experienced surgeons [15, 16]. One of the reasons for this is that the average number of fibroids extracted in GRP-LM is as high as 5 (median: 4). In a literature review of 23 facilities [17, 18], the mean or median number of fibroids extracted for conventional LM was between 1 and 2 (52%) in 12, between 2 and 3 (35%) in 8, between 3 and 4 (13%) in 3, and no facility had more than 4, and multiple fibroids (≥ 4) in different parts of the uterus increase the difficulty of surgery [19].

As shown in this study, a larger number of fibroids extracted results in more fibroids being enucleated and sutured, thus increasing the operative time.

However, the fact that there was no correlation between the number of fibroids extracted and the amount of blood loss suggests that rapid fibroid enucleation using strong traction force with a Tenaculum forceps and secure suturing, which are unique to the gasless surgery, contribute to reducing the amount of blood loss.

On the other hand, with regard to the long average hospital stay of 5.7 ± 5.1 days, LM is covered by health insurance in our country, and patients are discharged after careful observation of postoperative pain and fever pattern. Therefore, it is difficult to compare our results with those in the foreign literature.

As for complications, 2.08–11.1% have been reported in the literature [18], and 5.67% have been reported in a recent study of about 20,000 LMs in our country [20], so the 0.7% of complication rate of GRP-LM is very low. In addition, the rate of laparotomy transition was 0%, which may be attributed to the fact that the GRP-LM is capable of rapid response, such as suture ligation and aspiration. We also believe that this is due to the excellent operability of the forceps in the lifting technique.

On the other hand, surgeons performing *G*-LS find it difficult to establish a good operative field in obese patients [6]. Generally, in severely obese cases, the abdominal wall is thick, so even if the abdominal wall is lifted, the fat in the abdominal wall often hangs down and a good operative field is often not obtained. However, in this study, the effect was hardly observed. This is thought to be due to the fact that the average BMI of women is 22.6 in our country [21], which is very low compared to other countries [21, 22].

We were unable to make comparisons with the conventional LM due to the paucity of LM using pneumoperitoneum, so we were limited to a review of the literature. In addition, the number of cases for severe obesity was small and could not be adequately evaluated. In the future, it will be necessary to increase the number of cases for comparative study.

Although this study is a single-center study, we consider its surgical results to be sufficiently reliable, because it developed a technique that facilitates surgical procedures, such as fibroid enucleation and suture ligation, which are considered difficult in LM, using a subcutaneous abdominal wall lifting method and has been studied in a large number of cases. On the other hand, a limitation of this study is that the technique differs from conventional LM in many aspects, making a comparison between the two difficult.

## Conclusion

The GRP-LM is a suitable surgical method for myomectomy, because it allows powerful grasping and traction of the fibroid with the Tenaculum forceps, and rapid and reliable suture and ligation, despite having only one port for the procedure. In addition, it is economical due to the reduction of manpower and disposable products, as well as cosmetic aspects.

## Supplementary Information

Below is the link to the electronic supplementary material.

Figure S1 (A–I) Technique for securing operative field and method for port creation in gasless reduced-port laparoscopic myomectomy (GRP-LM) A: The Kirschner wire is inserted subcutaneously along the sagittal line of the median abdominal wall. B: The lifting handle chain is fixed to the lifting bar. C: A small incision of about 1.5 cm is made in the right lower abdominal wall. D: The ventral fascia was bluntly punctured to reach the peritoneum. E: The peritoneum is held with Pean forceps at two sites. F: The freed peritoneum is held with Pean forceps at four sites. G: A Lap Protector® is inserted into the abdominal wall aperture. H: Right lower abdomen with a Lap Protector® in place. I: A 5 mm trocar is inserted via the umbilical fossa under the surveillance of the endoscope. Supplementary file 3 (TIFF 3072 kb)

Figure S2 (A–F) Surgical procedure in gasless reduced-port laparoscopic myomectomy (GRP-LM). A: Appearance of a surgical operation with the suction tube and the electrocautery inserted into the Lap Protector.B: Appearance of the Lap Protector with the Tenaculum forceps, the suction tube and the electrocautery inserted simultaneously. C: Suturing of the uterine myometrium using a mechanical knot that is tied outside the body and ligated inside the body. D: Removal of fibroids from the abdominal cavity by fine cutting with a scalpelE: Removal of fibroids using a MemoBag® F: Massive intra-abdominal lavage (2,000~3,000ml) with physiological saline using a funnel.Supplementary file 4 (TIFF 3072 kb)

## Data Availability

The datasets used and analyzed during the current study available from the corresponding author on reasonable request.
