# Supplementary figures and images for: Factors affecting gasless reduced-port laparoscopic myomectomy (GRP-LM) using a subcutaneous abdominal wall lifting method: a retrospective analysis of a large cohort of 966 cases in Japan
Source: Arch Gynecol Obstet. 2024 Sep 9;311(2):375–83. doi: 10.1007/s00404-024-07706-9 (PMC11890381; doi:10.1007/s00404-024-07706-9)

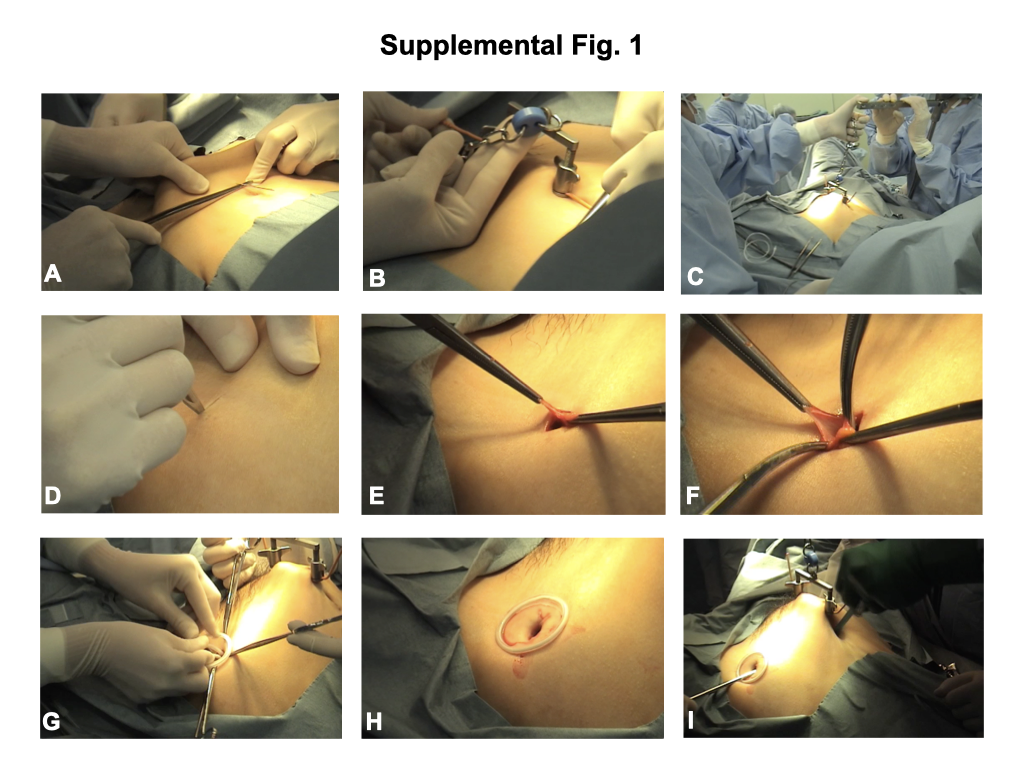

Supplement: Supplementary file 3 — Figure S1 (A–I) Technique for securing operative field and method for port creation in gasless reduced-port laparoscopic myomectomy (GRP-LM) A: The Kirschner wire is inserted subcutaneously along the sagittal line of the median abdominal wall. B: The lifting handle chain is fixed to the lifting bar. C: A small incision of about 1.5 cm is made in the right lower abdominal wall. D: The ventral fascia was bluntly punctured to reach the peritoneum. E: The peritoneum is held with Pean forceps at two sites. F: The freed peritoneum is held with Pean forceps at four sites. G: A Lap Protector® is inserted into the abdominal wall aperture. H: Right lower abdomen with a Lap Protector® in place. I: A 5 mm trocar is inserted via the umbilical fossa under the surveillance of the endoscope. Supplementary file 3 (TIFF 3072 kb) [file 404_2024_7706_MOESM3_ESM.tiff]

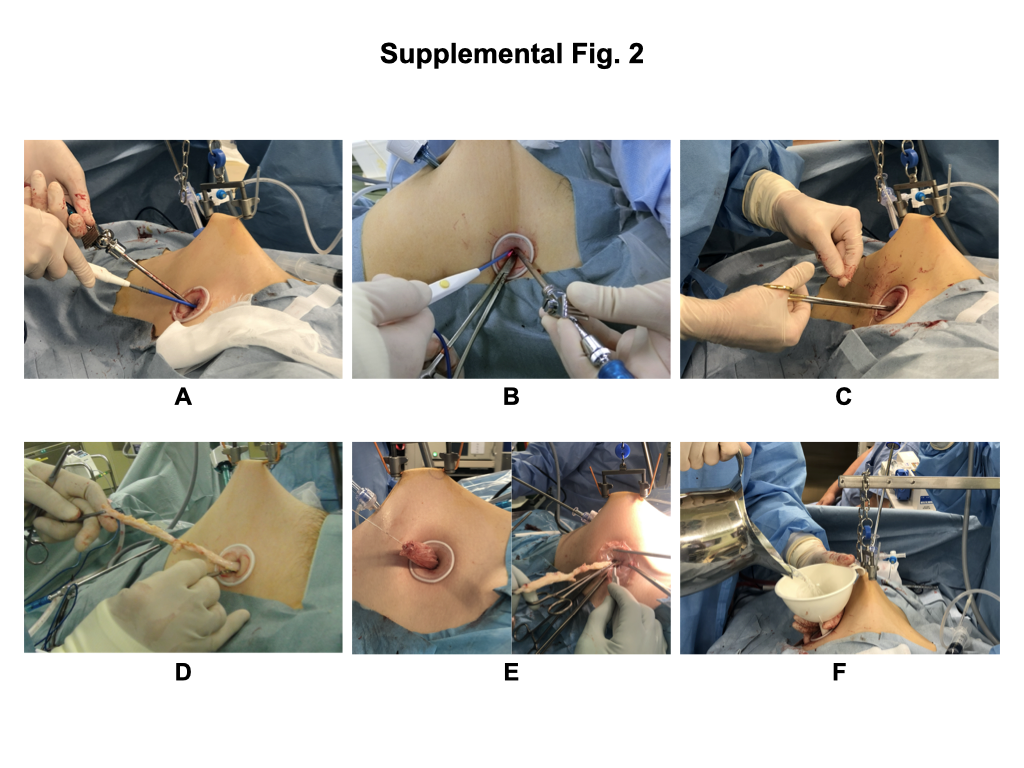

Supplement: Supplementary file 4 — Figure S2 (A–F) Surgical procedure in gasless reduced-port laparoscopic myomectomy (GRP-LM). A: Appearance of a surgical operation with the suction tube and the electrocautery inserted into the Lap Protector.B: Appearance of the Lap Protector with the Tenaculum forceps, the suction tube and the electrocautery inserted simultaneously. C: Suturing of the uterine myometrium using a mechanical knot that is tied outside the body and ligated inside the body. D: Removal of fibroids from the abdominal cavity by fine cutting with a scalpelE: Removal of fibroids using a MemoBag® F: Massive intra-abdominal lavage (2,000~3,000ml) with physiological saline using a funnel.Supplementary file 4 (TIFF 3072 kb) [file 404_2024_7706_MOESM4_ESM.tiff]
